# Can a virtual learning module foster empathy in dental undergraduate students?

**DOI:** 10.1111/eje.12783

**Published:** 2022-02-10

**Authors:** Sviatlana Anishchuk, Angela Kubacki, Yvonne Howell, Maria T. van Harten, Carilynne Yarascavitch, Caoimhin MacGiolla Phadraig

**Affiliations:** ^1^ Trinity College Dublin University of Dublin Dublin Dental University Hospital Dublin Ireland; ^2^ 4915 Institute of Medical and Biomedical Education St George's University of London London Ireland; ^3^ 7938 University of Toronto Toronto Canada

**Keywords:** clinical empathy, cognitive empathy, empathy, empathy in/ and curriculum, patient‐centred health care

## Abstract

**Background:**

Empathy is an essential part of patient‐centred health care, which positively benefits both patients and clinicians. There is little agreement regarding how best to design and deliver training for healthcare trainees to impart the skills and behaviours of clinical empathy. The study aimed to inform the field by sharing an educational intervention where we aimed to improve empathy amongst dental undergraduate students in Trinity College Dublin using a virtual learning module.

**Methods:**

Adopting pre–post‐repeat pre‐experimental design, dental professional students completed the Jefferson Scale of Empathy (JSE) for Health Professional Students immediately prior to and after a three‐week virtual programme designed to increase clinical empathy. Using a three‐factor model described for the JSE in the literature, scores were evaluated for internal consistency and paired tests were performed on scores appropriate to their distributions. Seven‐point Likert scales were scored to record student experience of training and technology, which are reported descriptively.

**Results:**

Most of the 37 participants were female (76%) and represented dental science (N = 27) and dental hygiene roles (N = 7). Results revealed a mean JSE‐HPS scale score rise from 110.0 (SD = 10.4) to 116.4 (SD = 11.1), which represented a rise of 5.8% (t (36) = 3.6, *p* = 0.001). The three factors associated with cognitive empathy, namely perspective‐taking (*T*(36) = 3.931, *p *< 0.001; *walking in the patient's shoes T*(36) = 2.093, *p* = 0.043); and compassionate care (*Z* = 2.469, *p* = 0.014) were all found to have increased after the intervention. Students reported a positive experience of discipline‐specific and generic videos as part of the module.

**Conclusion:**

The study demonstrated that a virtual educational module was associated with an increase in empathy amongst dental undergraduate students. The design of a blended module incorporating the Massive Open Online Course (MOOC) and virtual learning are beneficial and have a promising future.

## 1 INTRODUCTION

Empathy, as an emotion, can be difficult to define and deeply personal, having been proposed as “the almost magical emotion that persons or objects arouse in us as projections of our feelings and thoughts”.^1^ Moving beyond Spiro's question of “can empathy be taught?” to “how can empathy be fostered and maintained” has prompted healthcare educators to develop resources to keep empathy teaching in the curriculum. Clinical empathy is not simply “detached concern” but rather emotional atonement,^2^ and it describes the clinical skill of emotional resonance and curiosity about the meaning of a clinical situation for the patient.^3^ It is a clinical skill involving the active assessment of a patient's emotions and responding to patient cues.^4^ Most recently proposed as “empathic concern,” clinical empathy can be understood as “the attitude of genuine interest towards the experience of the other” which comes from an “engaged curiosity”.^5^

Clinical empathy is an effective means of enhancing patients’ experiences and outcomes of care. When practised appropriately, empathy builds rapport with the patient, facilitates the healthcare interview, increases the efficiency of gathering information in history taking and examination, increases adherence to treatment recommendations and health outcomes, and improves patient satisfaction.^6^, ^7^, ^8^ Unsurprisingly, empathic healthcare practitioners may also benefit through high work satisfaction, well‐being and low levels of burnout.^4^ Empathy is considered such an essential aspect of care^4^ that its absence impairs the clinician–patient relationship so much that it is associated with medical errors and other difficulties in care.^9^ Therefore, cultivating clinical empathy is one of the most important goals when training healthcare professionals.

Despite the obvious importance of empathy in health care, trainee health professionals consistently demonstrate a decline in empathy during their training.^10^, ^11^, ^12^ This seems to occur as students move into clinical years of their training, as trainees adapt to their professional roles^,^.^13^, ^14^ This may relate to the time when they begin seeing patients more frequently in their clinical years and demonstrate less metacognitive efforts.^15^ For dental students, it has been suggested that this decline could be attributed to a focus on the more technical demands of intensive training and the curriculum at dental schools being more procedurally focused rather than patient‐centred.^10^ Recognising the significance of this dramatic drop in empathy, healthcare curricula seek to impart empathy as a core attribute for the next generation of healthcare professionals.^16^ Whilst there is no agreed format with which to best foster empathy, there are three imperative factors that are believed to constitute empathy: “perspective‐taking,” “compassionate care” and “walking in the patients’ shoes” and these account for the development of a metacognitive side when interacting with patients.^17^

There are examples of good practice with various approaches, ranging from the use of an E.M.P.A.T.H.Y tool to improve doctor–patient communication,^18^ through to the use of video clips about patient encounters to achieve sustainable increases in empathy score amongst medical students.^19^

With no clear “best” way to foster clinical empathy, it is important to develop and evaluate approaches to impart empathy skills during health professional training. For this reason, we developed our undergraduate curriculum for dental care professional students (DCPs) to include a specific module on clinical empathy, which focused on how to recognise and understand the patient's feelings and experiences (perspective‐taking and walking in the patient's shoes) and how to communicate this understanding to their patients (compassionate care). With this in mind, our aim for this study was to assess whether completing this module could improve empathy. Within the context of the COVID‐19 pandemic, we designed and delivered a virtual learning module with this objective. We adopted a virtual experience primarily; this gave us the ability to maintain social distancing during learning. However, virtual learning also possesses many features that facilitate student learning. Online formats such as discussion boards help trainees participate on equal grounds, especially those reluctant to engage because they feel “socially awkward”.^20^ Greater involvement and engagement in peer learning and collaboration in virtual formats can contribute to deeper understanding and learning of the subject matter.^21^ The theoretical basis for this advantage can be drawn from social constructivism^22^ which is based on collaborative group work where knowledge is constructed through social interaction, and from experiential learning models, where learning is acquired through experience alone or by group, for example role play case studies.^23^ Moreover, utilising a community of inquiry framework, which represents a process of creating a deep and meaningful (collaborative constructivist) learning experience through the development of social, cognitive and teaching presence^24^ can enhance online learning and the student experience.

We aimed to evaluate the effectiveness of this educational intervention by assessing students’ empathy scores before and after training using the Jefferson Clinical Empathy Scale, with particular attention to the subtest scores. We also explored student perceptions of components of the intervention to explore the impact and acceptance of the disparate elements involved.

## 2 METHODS

### 2.1 Design

A pre‐experimental educational evaluation was carried out using repeat measures immediately before and after a two‐week training module aiming to investigate, first, changes in student's empathetic responses and second, effectiveness of the empathy training educational intervention. The evaluation was non‐controlled and non‐blinded and delivered to undergraduate DCPs without previous empathy training. The Dental School Research Ethics Committee of Trinity College Dublin granted ethical approval for this study in February 2021.

### 2.2 Sample

In April 2021, dental students who were enrolled in undergraduate inter‐professional learning at the School of Dental Science in Trinity College Dublin were invited to participate in the study (N = 62). Research participants included fourth‐year dental, second‐year dental hygiene and second‐year dental nursing students.

### 2.3 Intervention description

The virtual empathy module included two elements: asynchronous (two hours) and synchronous (two hours), distributed over three weeks (see Table 1).

**TABLE 1:** Learning Plan for Empathy Module

	Session Learning Outcome(s)	Delivery	Element
Week 1 Element 1 Step 1	Describe the material to be covered in the module, the learning outcomes and trainers. Access training resources.	Virtual synchronous introduction	Synchronous
	Complete Baseline survey online.		Synchronous
	Discuss the definition of empathy	MOOC	Asynchronous
	Describe the difference between empathy and sympathy	MOOC	Asynchronous
	Explore the use of emotional and cognitive empathy	MOOC	Asynchronous
	Explore the importance of empathy in health care, MOOC Text summarising evidence (short)	MOOC Discussion forum	Asynchronous
Week 2 Element 1 Step 2	Identify opportunities for empathic behaviour	MOOC	Asynchronous
	Reflect on verbal and non‐verbal empathic responses	MOOC	Asynchronous
	Discuss the challenges of being empathic as a healthcare professional	MOOC	Asynchronous
	Identify the importance of self‐awareness and empathic practice	MOOC Text summarising Evidence (short) Discussion forum	Asynchronous
Week 3 Element 2	Identify empathetic opportunities in the dental context Video 1 www.youtube.com	Video 1 Watch video and note missed empathetic opportunities	Synchronous
	Reflect on verbal and non‐verbal empathetic response in the dental context Video 2 www.youtube.com	Video 2 Watch video and note empathetic responses	Synchronous
	Discuss the challenges of being empathetic as an oral healthcare professional	Discussion in break out rooms Note challenges of empathetic practice	Synchronous
	Review and discuss learning outcomes and future practice	Synchronous discussion Formulate strategies for future practice	Synchronous
		Survey online Complete the Jefferson scale 2 post‐test	Synchronous

### 2.4 Element 1: Virtual Asynchronous

Following an initial tutorial about the programme, the Element 1 was delivered through a Massive Open Online Course (MOOC) entitled “Developing Clinical Empathy: Making a Difference in Patient Care,” which is aimed at all healthcare professionals and trainees. The MOOC content was designed by a team of Clinical Communication experts, to enhance the learners’ ability to better recognise and understand the patient's perspective, communicate empathically and demonstrate compassionate care. This is a compulsory component of the clinical communication curriculum for the undergraduate medicine course at St George's, University of London, UK.^25^ It is open access and free to join for all learners on the FutureLearn platform. This element involved two steps.

Step one provided an exploration of the definition, meaning and importance of empathy in health care, followed by a case study that asked participants to consider how they would respond to a patient in distress. Participants were encouraged to reflect on their own experiences of empathic communication and how they recognise empathy from others. In Step two of Element 1, participants learned how to identify and respond to empathic opportunities by recognition of both verbal and non‐verbal cues. Challenges to empathic communication and skills for maintaining empathy in tele‐consultations were also addressed. The course provided access to recommended reading, videos, audio recordings, and includes short quizzes to test knowledge and cognitive learning.

Participants were encouraged to reflect, ask questions and post comments for other learners in the discussion board moderated by healthcare educators. Utilising a collaborative‐constructivist approach, discussion activities within the MOOC encourage learners to construct personal meaning through their engagement with the material by sharing personal experiences and challenges within their own empathic practice, thus forming a “community of inquiry” group.^26^ Trained moderators provided the “teacher presence,” which has been associated with learner satisfaction and engagement.^27^

### 2.5 Element 2: Virtual Synchronous

The synchronous element was specifically designed for dental professionals to transfer general concepts introduced in the MOOC to the dental context and their future practice. This element bookended the module and included an introductory session at the beginning and was followed two weeks later by an interactive session after students completed Element 1 (MOOC). This provided a content overview, specific dental video scenarios and discussion groups (Table 1). The learning outcomes for this section were designed specific to the dental context and are listed in Table 1 week 3.

### 2.6 Data Collection

Participants completed an anonymous online survey immediately before and after the module. Attendees were advised that the data would serve primarily as a course evaluation but were asked to specifically opt‐in to have their responses included in this research study.

This survey consisted of (1) demographic information, (2) experience of specific components of training scored on a seven‐point Likert scale and (3) Jefferson Scale of Empathy for Health Professional Students (JSE‐HPS).^28^ The JSE‐HPS consists of 20 items answered with 7‐point Likert scale (Strongly Disagree 1 and Strongly Agree 7), where 10 items are positively worded (directly scored), and 10 items are negatively worded (reverse scored) with a range from 20 to 140. The Health Professionals Student Version (HPS‐version) is oriented at clinical students other than medical students.^13^, ^29^, ^30^ The content of the questionnaire addresses three factors: perspective‐taking “view of patients perspective” and “emotions in patient care” (10 items), compassionate care “understanding patient's experiences” (8 items) and walking in patient's shoes “thinking like the patient” (2 items). The “perspective‐taking” has been described as the central cognitive ingredient of empathy and empathetic engagement.^31^ The second factor “compassionate care” describes the importance of the patient conveying his or her emotion, and the practitioner recognising and translating these feelings. The third factor “walking in patient's shoes” expresses a view of the situation from the patient's perspective.^31^

Post‐module survey additionally included a set of statements on experience of content delivery, learning activities and technology use regarding the MOOC element of training and their value to the training. This is described in more detail in the course feedback section.

### 2.7 Data analysis

Data were analysed using IBM SPSS 25. JSE‐HPS scores were computed per JSE instructions.^17^ Shapiro–Wilk tests, as recommended by Ghasemi and Zahediasl,^32^ and histograms were used to judge the normality of the difference between the scores by the factors perspective‐taking, compassionate care and walking in patient's shoes. Furthermore, Cronbach's alpha was calculated for each set of scores. Several studies have confirmed that this three‐factor model is appropriate for the JSE using confirmatory factor analysis.^17^ Paired tests were carried out appropriate to the distributions. Effect sizes for non‐parametric data were calculated from original means and standard deviations of the related samples to compute Cohen's *d* ^33^ and Rosenthal's^34^ method for *r*. Mean, standard deviation of summative scale scores were calculated at baseline and after the training. Cronbach's alpha was used as a measure of internal consistency. Pre‐ and post‐summative scores were compared using related samples Student's t‐test.

## 3 RESULTS

### 3.1 Sample description

Thirty‐seven students consented and participated in both the pre‐ and post‐repeat surveys (Participation Rate = 59.7%). Thirty‐five participants provided their age: the range was 19 to 33, with a mean age of 23.1 (*SD* 3.1). Twenty‐eight of 37 participants (75.7%) were female, and 27 (73.0%) were Dental Science students (Table 2).

**TABLE 2:** Characteristics of the sample

	Count (n)	Per cent (%)
Total	37	100
Gender		
Male	9	24.3
Female	28	75.7
Current undergrad training		
Dental nurse	3	8.1
Dental hygiene	7	18.9
Dental science	27	73.0

### 3.2 Pre–post‐measures

At baseline, the JSE‐HPS scores ranged from 84.0 to 135.0 with a mean score of 110.0 (SD 10.4). After training, the scores ranged from 81.0 to 135.0 with a mean score of 116.4 (SD 11.1) (Table 3). Internal consistency of the pre‐ and post‐scores was measured using Cronbach's alpha^35^; these were 0.74 and 0.78, respectively, which are acceptable. The difference between post‐ and pre‐scores was also calculated, and its distribution was summarised. When pre‐ and post‐scores were compared using a paired Student's t‐test, they were found to be different with a mean difference of 6.4 (t (36) = 3.6, *p* = 0.001) or 5.8% and effect size d = 0.59 (Table 4).

**TABLE 3:** Characteristics of the JSE‐HPS Scores

	Mean	Std dev	Cronbach's alpha
Pre‐score	110.0	10.4	0.74
Post‐score	116.4	11.1	0.78
Δ _post‐score–pre‐score_	6.4	10.8	

**TABLE 4:** Descriptive statistics, internal consistency of scores and comparison of scores by factor

Factor	Mean	SD	Cronbach's alpha	Paired test statistic	Paired test p‐value	Cohen's d	Effect size r
Perspective‐Taking							
Pre	58.9	6.0	0.70				
Post	62.6	5.5	0.78				
Δ Post–Pre	3.73	5.8		T(36) = 3.931	<0.001	0.64	0.31
Compassionate Care							
Pre	43.9	5.1	0.49				
Post	45.5	8.0	0.82				
Δ Post–Pre	1.6	7.7		Z = 2.469	0.014		0.41
Walking in Patient's Shoes							
Pre	7.2	2.5	0.78				
Post	8.3	3.1	0.88				
Δ Post–Pre	1.1	3.1		T(36) = 2.093	0.043	0.39	0.19

The Cronbach's alpha values for each set of scores by factor are good overall, suggesting that the questions comprising each of the three factors are unidimensional.

Paired *t*‐tests performed on the normally distributed perspective‐taking and Walking in patient's shoes post‐subtracted by pre‐intervention data showed that the interventions elicited statistically significant changes in these two factors (*T*(36) = 3.931, *p* < 0.001; *T*(36) = 2.093, *p* = 0.043). The intervention had a moderate effect in perspective‐taking (*d* = 0.64, *r* = 0.31) and a small effect in walking in patient's shoes (*d* = 0.39, *r* = 0.19), using Cohen's (1988)^33^, ^36^ effect size conventions.

A Wilcoxon signed‐rank test performed on the non‐normally distributed compassionate care post‐subtracted by pre‐intervention data showed that the intervention elicited a statistically significant change in the factor compassionate care (*Z* = 2.469, *p* = 0.014). Indeed, median scores increased from 44.0 to 47.0. The effect was moderate (*r* = 0.41) using Cohen's (1988)^33^, ^36^ effect size conventions.

### 3.3 Course feedback

**FIGURE 1:**
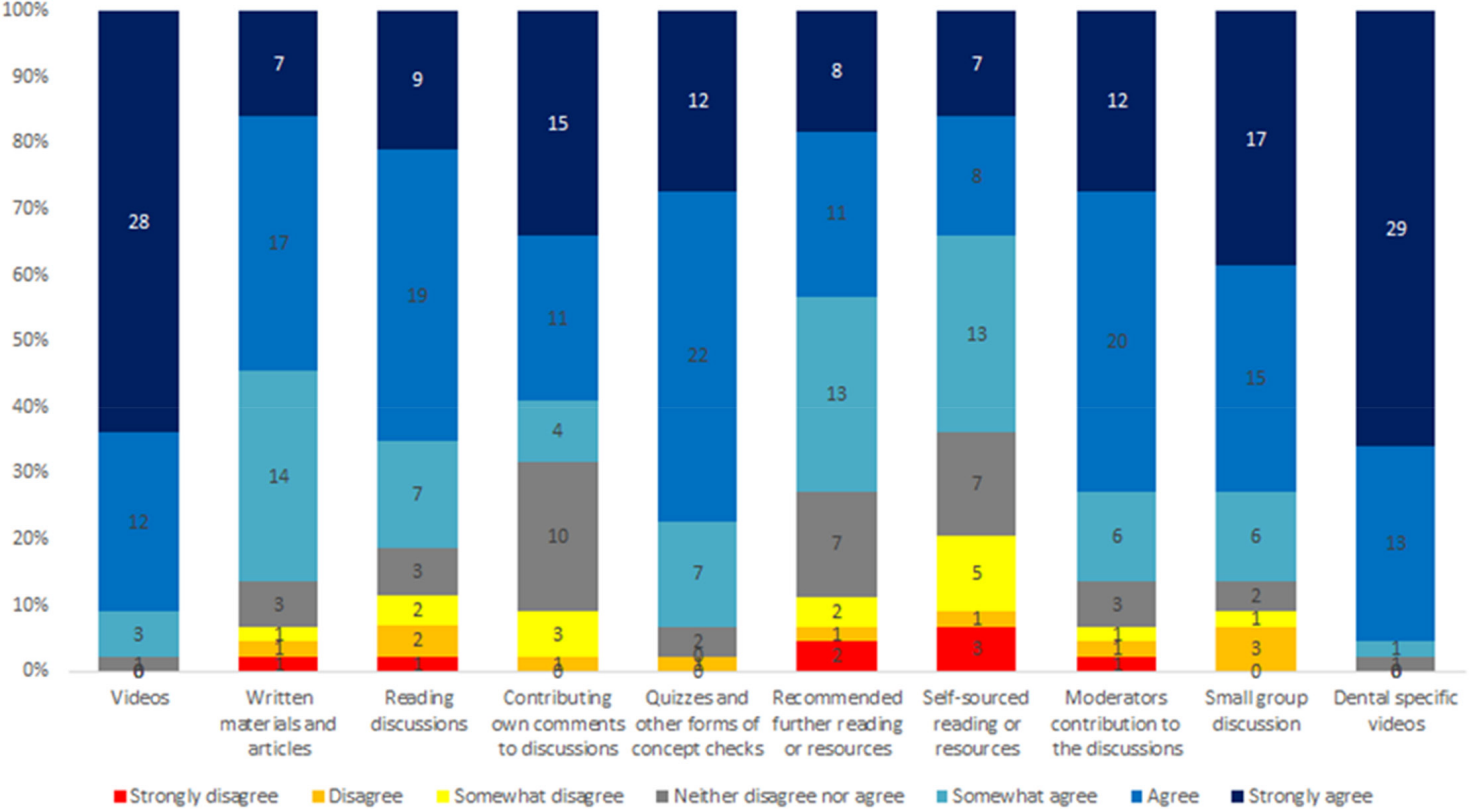
Experience of content delivery and learning activities. Feedback question: How strongly do you agree that the activities below added value to your own learning?

**FIGURE 2:**
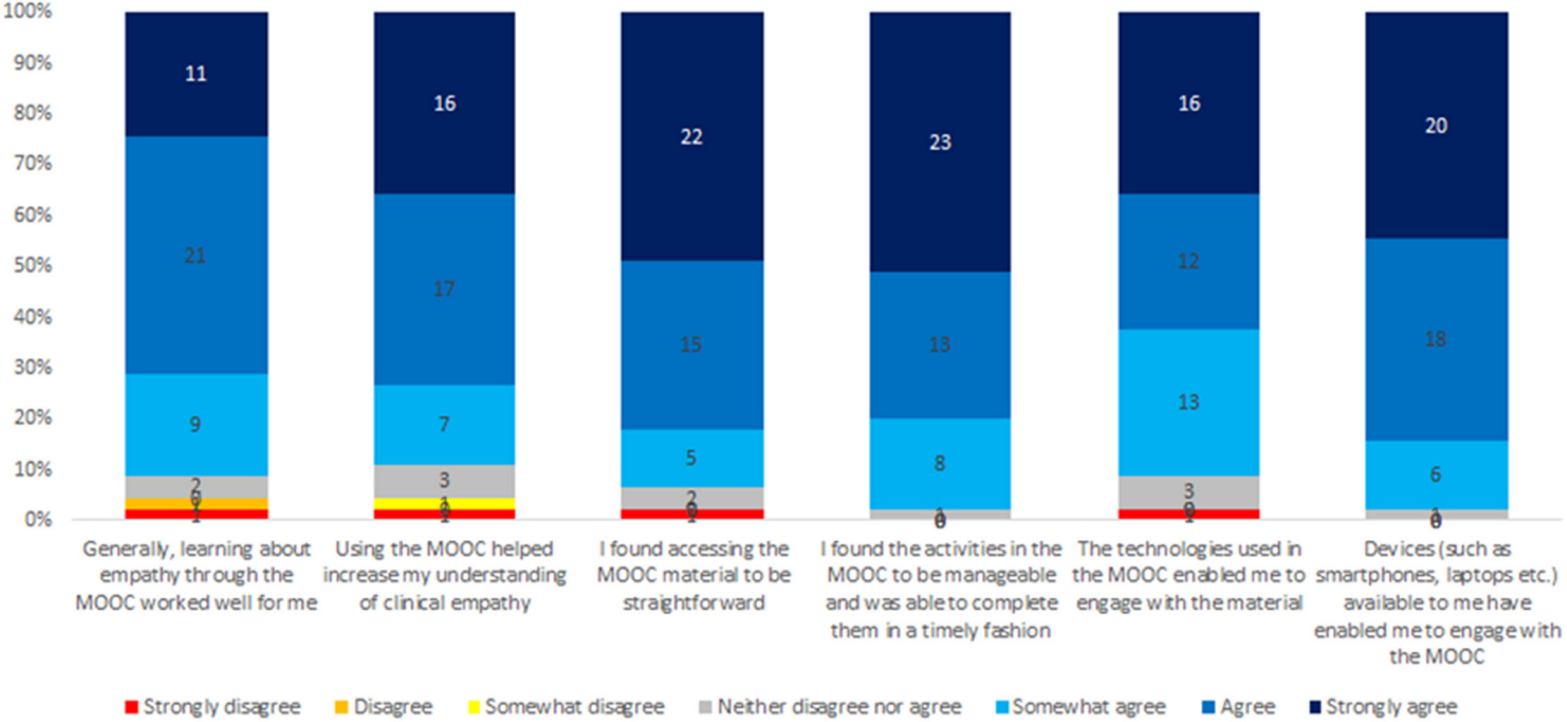
Experience and technology use regarding MOOC element of training. Feedback question: Indicate the extent of your agreement or disagreement with each of the following statements

With respect to the value of learning activities, students were most likely to agree that videos added value, particularly dental‐specific videos, whereas reading articles was least likely to add value (Figure 1). The majority of students (N = 38, 84.5%) reported completing the MOOC in full, with a further 4 (8.8%) completing either half or three‐quarters of it. Forty‐three (95.6%) reported they would recommend the MOOC to other healthcare professionals and colleagues. Further feedback on the use of technology and experience of the MOOC element of training is illustrated in Figure 2.

## 4 DISCUSSION

This study intended to measure change in empathy in dental professional students before and after a bespoke virtual training module, as well as the effectiveness of the virtual module as an educational intervention. Overall, the study found a rise in JSE‐HCP scores of 5.8% by the end of training. More specifically, the three factors associated with cognitive empathy: “perspective‐taking,” “compassionate care” and “walking in the patients’ shoes,”^37^ have increased on post‐interventions *p* < 0.001, *p* = 0.014 and *p* = 0.043 respectively. These findings suggest that the virtual training module fostered clinical empathy. Our findings are consistent with past literature that describes modest increases in empathy scores amongst various disciplines such as pharmacy, biomedical science, radiography, radiation therapy, nursing, paramedics and medicine students following educational interventions.^11^, ^19^, ^38^

The virtual learning module in this study adopted a blend of learning technologies, including online case‐based discussion and role‐modelling through videos specifically scripted to promote perspective‐taking and an enhanced understanding of the patient's experience and feelings. Whilst students rated all aspects and media as valuable to their learning, videos (general medical and dental‐specific) were most often considered valuable, followed by discussion groups, whereas the self‐sourced reading was least often reported as valuable. This may be because observing patient's reactions and body language as well as listening to their story does help students consider how they might respond to that patient and how they themselves are feeling about the patient's situation. On one hand, this is in line with the results from other studies where DVD‐based workshops and video clips improved the empathy level amongst students.^11^, ^19^ The research around the integration of MOOCs in traditionally taught courses continues to grow, and evidence suggests that learning outcomes can be achieved successfully using a blended approach.^39^ On the other hand, it is not surprising that the preferred learning methods were medical and dental‐specific videos. Williams et al. and Hojat at el.^11^, ^40^ found that educational interventions tailored towards specific professions were also preferred in their studies. Although empathy is a complex phenomenon amongst different professions, it was suggested that training should be amenable and routinely integrated into curricula^11^and that the intervention required to increase clinical empathy can be cost‐effective and can be delivered at any point in the curriculum.

These findings overall support the integration of our elements including a MOOC into bespoke modules and promote the interdisciplinary production of learning modules between communication and dental experts.

## 5 LIMITATIONS AND STRENGTHS

Our study is limited by design, single post‐test and sample size. The lack of a control group limits our ability to directly attribute outcomes to the training. It was not possible to generate a control group from the convenience sample within the chosen educational framework. The long‐term stability of the observed changes is unknown, as only a single post‐test was conducted. The small sample size within a single institution also limits inferences. This smaller sample was partially due to our decision to only include those with matched data, which allowed for more conservative analyses of paired data with increased precision. The main strengths of this study come from the robust intervention and evaluation. The intervention used a multidisciplinary approach in development and incorporated a blend of virtual educational technologies. The use of a validated tool and the demonstration of acceptable internal consistency within our sample suggests that our outcomes are reliable.

### 5.1 Implications of our findings

This study provides useful information that may benefit educators for both practice and research. Our findings suggest that training in clinical empathy using a virtual module may improve clinical empathy in the short term amongst dental professional students. The results of this study indicate that video vignettes with scenarios that encourage perspective‐taking for empathic skill training, such as identifying opportunities for empathic engagement and appropriate responses, had the highest value for trainees. Although general scenarios were valued, some thought should be given to provide context‐specific scenarios in order to maximise learning impact. Readers are invited to consider our videos as exemplars or for use in their own programmes (Developing Clinical Empathy ‐ Online Course – FutureLearn; Video 1 – YouTube; Video 2 – YouTube). Regarding research, our use of the Jefferson scale is an additional demonstration of internal reliability amongst dental professional students. Future research that incorporates a control group, head‐to‐head comparisons of alternative learning interventions and randomisation with delayed post‐test will help to further define learning impact.

## 6 CONCLUSION

A virtual educational module to teach clinical empathy, incorporating a MOOC and bespoke training materials, was associated with an increase in empathy scale score amongst dental care professional students. These results provide evidence for the advocating inclusion of empathy training in dental education. The results suggest that a virtual module incorporating MOOCs is beneficial and have a promising future in helping healthcare students to sustain empathy scores for longer time periods. More research is needed to increase confidence in this assumption and understand which element of training is most influential in improving cognitive empathy.

## CONFLICT OF INTERESTS

The authors have no conflicts of interest to disclose.

## Data Availability

Research data are not shared.
